# Association between *Prostinogen (KLK15)* Genetic Variants and Prostate Cancer Risk and Aggressiveness in Australia and a Meta-Analysis of GWAS Data

**DOI:** 10.1371/journal.pone.0026527

**Published:** 2011-11-23

**Authors:** Jyotsna Batra, Felicity Lose, Tracy O'Mara, Louise Marquart, Carson Stephens, Kimberly Alexander, Srilakshmi Srinivasan, Rosalind A. Eeles, Douglas F. Easton, Ali Amin Al Olama, Zsofia Kote-Jarai, Michelle Guy, Kenneth Muir, Artitaya Lophatananon, Aneela A. Rahman, David E. Neal, Freddie C. Hamdy, Jenny L. Donovan, Suzanne Chambers, Robert A. Gardiner, Joanne Aitken, John Yaxley, Mary-Anne Kedda, Judith A. Clements, Amanda B. Spurdle

**Affiliations:** 1 Australian Prostate Cancer Research Centre-Queensland and Institute of Health and Biomedical Innovation, Queensland University of Technology, Brisbane, Queensland, Australia; 2 Molecular Cancer Epidemiology, Queensland Institute of Medical Research, Brisbane, Queensland, Australia; 3 Statistics Unit, Queensland Institute of Medical Research, Brisbane, Queensland, Australia; 4 School of Nursing and Midwifery, Queensland University of Technology, Brisbane, Queensland, Australia; 5 Section of Cancer Genetics, The Institute of Cancer Research & Cancer Genetics Unit, The Royal Marsden NHS Foundation Trust, London and Surrey, United Kingdom; 6 Cancer Research UK Genetic Epidemiology Unit, Strangeways Laboratory, Cambridge, United Kingdom; 7 Health Sciences Research Institute, Warwick Medical School, Warwick University, Coventry, United Kingdom; 8 Division of Epidemiology and Public Health, University of Nottingham, Queens Medical Centre, Nottingham, United Kingdom; 9 Department of Oncology, University of Cambridge, Cambridge, United Kingdom; 10 Nuffield Department of Surgical Sciences, John Radcliffe Hospital, Oxford, United Kingdom; 11 School of Social and Community Medicine, University of Bristol, Canynge Hall, Bristol, United Kingdom; 12 Griffith Health Institute, Griffith University, Brisbane, Queensland, Australia; 13 Viertel Centre for Research in Cancer Control, Cancer Council Queensland, Brisbane, Queensland, Australia; 14 University of Queensland Centre for Clinical Research, Royal Brisbane Hospital, Brisbane, Queensland, Australia; 15 Brisbane Private Hospital, Brisbane, Queensland, Australia; Federal University of São Paulo, Brazil

## Abstract

**Background:**

*Kallikrein 15 (KLK15)/Prostinogen* is a plausible candidate for prostate cancer susceptibility. Elevated *KLK15* expression has been reported in prostate cancer and it has been described as an unfavorable prognostic marker for the disease.

**Objectives:**

We performed a comprehensive analysis of association of variants in the *KLK15* gene with prostate cancer risk and aggressiveness by genotyping tagSNPs, as well as putative functional SNPs identified by extensive bioinformatics analysis.

**Methods and Data Sources:**

Twelve out of 22 SNPs, selected on the basis of linkage disequilibrium pattern, were analyzed in an Australian sample of 1,011 histologically verified prostate cancer cases and 1,405 ethnically matched controls. Replication was sought from two existing genome wide association studies (GWAS): the Cancer Genetic Markers of Susceptibility (CGEMS) project and a UK GWAS study.

**Results:**

Two *KLK15* SNPs, rs2659053 and rs3745522, showed evidence of association (p<0.05) but were not present on the GWAS platforms. *KLK15* SNP rs2659056 was found to be associated with prostate cancer aggressiveness and showed evidence of association in a replication cohort of 5,051 patients from the UK, Australia, and the CGEMS dataset of US samples. A highly significant association with Gleason score was observed when the data was combined from these three studies with an Odds Ratio (OR) of 0.85 (95% CI = 0.77–0.93; p = 2.7×10^−4^). The rs2659056 SNP is predicted to alter binding of the RORalpha transcription factor, which has a role in the control of cell growth and differentiation and has been suggested to control the metastatic behavior of prostate cancer cells.

**Conclusions:**

Our findings suggest a role for *KLK15* genetic variation in the etiology of prostate cancer among men of European ancestry, although further studies in very large sample sets are necessary to confirm effect sizes.

## Introduction

Prostate cancer is the most common cancer (after skin cancers) in the Western world with one in nine men expected to develop prostate cancer by the age of 75 and 20,000 new cases being diagnosed annually in Australia (Prostate Cancer Foundation of Australia, http://www.prostate.org.au, 2010). Age, race and family history of prostate cancer are well-established risk factors for prostate cancer [1]. In addition, there is considerable evidence for a genetic basis underlying risk for prostate cancer [2], [3]. The chromosomal region 19q12-13 is of considerable interest, as previous gene and protein expression studies have shown this region to harbor both prostate cancer susceptibility and aggressiveness loci [4], [5], [6], [7]. The human *Kallikrein* (*KLK*) gene family comprises 15 genes and is clustered together in a small region of approximately 320 kb at chromosome 19q13.4 [7], [8], [9]. *KLK15* (also called Prostinogen) is the most recently cloned member of the human *Kallikrein* gene family and is adjacent to *KLK3/prostate specific antigen (PSA)* in genetic location [10], [11]. *KLK15* has been reported to be upregulated at the mRNA level in prostate cancer [11], [12], [13] and has been described as an unfavorable prognostic marker for prostate cancer progression following radical prostatectomy [14]. *KLK15* has also been reported to be a significant predictor of reduced progression-free survival and overall survival in ovarian cancer [15] and a favorable prognostic marker for breast cancer [16].

Studies of *KLK* genetic variants and their association with cancer have increased in the last few years with the aim to better understand the biology of cancers and to develop potential new targets for genetic testing with regard to cancer risk and prognostic value [6], [10], [17], [18], [19], [20], [21], [22]. Recently, genome-wide association studies (GWAS) have identified a number of single nucleotide polymorphisms (SNPs) that are associated with risk of developing prostate cancer. One of these hits, rs2735839, is close to the *KLK3* (PSA) gene [23], [24]. There is some debate as to whether the SNP is associated with prostate cancer or simply correlates with PSA expression levels, as controls used for stage 1 of this GWAS were limited to those with low PSA levels (<0.5 ng/ml) [19], [23]. However, these results were replicated in studies with PSA unselected controls, including our study group [23], signifying the importance of this region in prostate cancer. Specifically since *KLK15* is located adjacent to *KLK3*, and shows altered expression in prostate cancer, it is a very plausible candidate prostate cancer gene.

Although some *KLK15* SNPs have been genotyped in GWAS, the large majority of variation in the *KLK15* gene remains unexplored for an association with prostate cancer. Investigation of a number of public databases, including NCBI Entrez-dbSNP (http://www.ncbi.nlm.nih.gov/entrez/db=snp), reveals the above-mentioned GWAS platforms cover from approximately 6–55% of validated variation in the *KLK* genes (Lose, Batra *et al*, unpublished data, 2010). These observations prompted us to undertake an association study between twenty-two *KLK15* SNPs, identified through *in silico* and sequencing approaches, with the risk of prostate cancer in a large group of Australian men with prostate cancer and male controls not screened for PSA levels. SNPs found to be associated with prostate cancer risk and/or aggressiveness were also assessed using GWAS data from additional replication datasets in the UK, Australia [24] and USA [25].

## Results

### *In silico* analysis, *KLK15* promoter sequencing and Linkage Disequilibrium mapping

We have used *in silico* prediction of function of wildtype and variant promoter sequences through assessment of hormone receptor elements and transcription factor binding sites; as well as prediction of likely splice variants through genomic, splicing and EST databases and web sites, and multiple sequence alignment packages as described previously [26]. We sequenced germline DNA from 20 aggressive prostate cancer patients (Gleason score >7) within the putative *KLK15* promoter and detected 20 SNPs (6 of which were classified as not validated by NCBI database at the time of data generation). Seven non-validated SNPs from the NCBI database were found to be non-polymorphic in our sequencing cohort. Further, we identified two novel SNPs, but neither was predicted *in silico* to have a functional role and hence was not considered for further genotyping.

SNPs chosen for genotyping in this study were (i) identified as tagging SNPs using HapMap version 22 (April 07), using a minor allele frequency >0.05 and pairwise linkage disequilibrium threshold of r^2^>0.8 (rs2659058, rs3212810, rs3745522, rs2659056, rs266851, rs2163861, rs266856), or (ii) chosen due to the *in silico* prediction of a functional effect on *KLK15* expression (rs3212853, rs3212852, rs16987576, rs2659055, rs266853, rs266854, rs190552, rs266855, rs2739442, rs2033496, rs12978902, rs2659053, rs2569746, rs35711205, rs2569747) (Table S1). As the frequency data for many of these SNPs was not available, we genotyped all 22 SNPs in >1000 male controls and generated a linkage disequilibrium (LD) map using Haploview 4.2 (Figure S1). All SNPs except rs3745522 were found to follow Hardy–Weinberg Equilibrium (p<0.01) (Table S1). SNP rs12978902 was non-polymorphic, while rs3212853, rs3212852, rs16987576, rs266853 and rs266854 were found to have minor allele frequencies <0.05 (Table S1), so were not pursued further for the association analysis. SNPs in high LD with other SNPs (r^2^>0.9; rs2163861, rs266856, rs2033496, rs2569747) were also not analysed further. Priority was given to putative functional SNPs, with a total of 12 SNPs shortlisted for further genotyping in Australian prostate cancer patients and controls (Table S1).

### Association with prostate cancer and disease aggressiveness

Initially DNA from 1,011 men recently diagnosed with prostate cancer and 1,405 male controls from Queensland (QLD), Australia, were analysed in this study. Table 1 illustrates a number of the socio-demographic and clinical characteristics of the QLD sample set studied. For specific SNPs where replication was sought, a maximum of 10,685 prostate cancer cases and 12,515 matched controls from UK, Australia and USA were included in the study.

**Table 1:** Socio-demographic and clinical characteristics of the QLD study populations.

Characteristics	Men with prostate cancer (*n* = 1011) *n* (%)	Healthy controls (*n* = 1405) *n* (%)
Age in years (median, range)	64 (43–88)	62 (18–75)
BMI (Mean, SD)	24.3 (11.6)	26.5 (7.3)
Marital status		
Never married	40 (4)	101 (7)
Married/de facto	847 (84)	1124 (80)
Divorced/separated/widowed	118 (11)	161 (12)
Unknown	6 (1)	25 (1)
Family history of prostate cancer^a^		
No	722 (72)	1253 (89)
Yes	286 (28)	151 (11)
Vasectomy status^b^		
No	109 (72)	847 (62)
Yes	43 (28)	521 (38)
Smoking status		
Never smoked^c^	404 (40)	596 (42)
Former smoker	527 (52)	751 (54)
Current smoker	78 (8)	37 (3)
Unknown	2 (0)	21 (2)
Alcohol consumption^b^		
Non-drinker	57 (38)	180 (13)
Drinker	95 (62)	1207 (87)
Highest education level achieved		
No formal education/primary school	139 (14)	245 (17)
Secondary school	361 (36)	330 (24)
Technical college	321 (32)	447 (32)
University	180 (17)	365 (26)
Unknown	10 (1)	18 (1)
Self reported Serum PSA levels		
<4 ng/ml	119 (12)	Not measured
4–10 ng/ml	526 (52)	Not measured
>10 ng/ml	249 (24)	Not measured
Unknown	126 (12)	Not measured
Gleason score (Gleason grade 1+Gleason grade 2)		
<7	231 (23)	Not applicable
≥7	559 (55)	Not applicable
Unknown	221 (22)	Not applicable

a positive family history is defined as at least one first degree relative with prostate cancer.

b Data was not collected for the retrospective study. Study characteristics differed significantly between cases and controls (*P*<0.01).

c Smokers are people who smoked at least 100 cigarettes in their entire life.

When twelve of the *KLK15* SNPs were assessed individually (Table S2) in the Australian sample set, two were found to be marginally associated with risk of prostate cancer (Table 2), neither of which has data available from existing UK GWAS and CGEMS sample sets. The age adjusted OR for rs2659053 was 1.25 (95% CI = 1.04–1.50; p = 0.050) for the GA genotype compared to the wildtype GG genotype. The CG genotype of rs35711205 displayed an OR of 1.27 (95% CI = 1.06–1.52; p = 0.027) compared to the common CC genotype (Table 2). To obtain more comparable age distributions, we reanalyzed our data excluding all controls younger than the youngest case (i.e. all controls less than 43 years, N = 70) and similar results were obtained for both of these SNPs (rs2659053: OR = 1.25, 95% CI = 1.05–1.51, rs35711205: OR = 1.28, 95% CI = 1.07–1.53). We also observed a similar result when case-control analysis was restricted to Caucasian subjects (data not shown) or when analyses included aggressive patients only (Gleason score≥7) (Supplementary Table S2). These SNPs were not found to be significantly associated with prostate cancer risk in a recently published study, where results were imputed from nextgen sequencing data and PLCO study group from CGEMS dataset, Table 2 [27].

**Table 2:** Association between *KLK15* SNPs and prostate cancer risk in the QLD and PLCO study groups.

Qld DATA					PLCO Cancer Screening Trial (CGEMS)	
SNP^a^	Control	Cases	OR (95% CI)	P value^b^	OR (95% CI)	p value^c^^,^ d
*rs2659053*						
GG	542 (39.5)	348 (35.0)				
GA	615 (44.9)	486 (48.8)	1.25 (1.04–1.50)	0.050	1.02 (0.83–1.25)	0.839
AA	214 (15.6)	161 (16.2)	1.19 (0.92–1.52)			
*rs35711205*						
CC	914 (66.0)	618 (61.3)				
CG	410 (29.6)	350 (34.7)	1.27 (1.06–1.52)	0.027	1.16 (0.90–1.49)	0.253
GG	60 (4.3)	40 (4.0)	0.96 (0.63–1.46)			

a SNP identifier based on NCBI dbSNP; SNPs are included in the region of the *KLK15* gene including 2 kb of transcription start sites.

b The result of 2-d.f. test based on logistic regression in the Queensland study adjusted for age as continuous variable.

c Imputed from 1000 Genomes project data and PLCO genotyped data, where actual genotype data not available, [27]; allelic OR and p values are presented.

d The result of 2-d.f. test based on logistic regression in the PLCO study adjusted for age in five-year intervals, study center, and three eigenvectors to control population stratification in an incident density sampling strategy.

*KLK15* SNP rs2659056 was found to be associated with risk of prostate cancer in UK stage 1 GWAS only, with OR = 2.01 (95% CI = 1.50–2.68; p = 5.45×10^−7^), but was not found to be significantly associated with prostate cancer risk in the QLD dataset (OR = 1.16, 95% CI = 0.83–1.62; p = 0.41) or the PLCO study group from CGEMS dataset (OR = 0.95, 95% CI = 0.68–1.33; p = 0.94) (Table S2).

Analysis of the association of rs2659056 with Gleason scores using case-case analysis of the QLD dataset revealed a significant association (Table 3). The C allele was significantly more common in patients with less aggressive disease compared to patients with more aggressive disease with per allele OR = 0.70, 95% CI = 0.56–0.89; p = 0.003) (Table 4). Analysis of this SNP in the available replication sets showed evidence for association in the UK stage 3 dataset (OR = 0.87, 95% CI = 0.78–0.98; p = 0.020) and the results were in the same direction for the CGEMS dataset (OR = 0.93, 95% CI = 0.77–1.12; p = 0.43) but not the other 2 studies (Table 4; Figure S2). The combined estimates for all 5 studies was OR = 0.92 (95% CI = 0.86–0.98), but with significant evidence of heterogeneity (p = 0.023). The heterogeneity of the ORs was minimized when we restricted our combined analysis to the QLD, UK GWAS stage 3 and CGEMS datasets (heterogeneity p = 0.86). Utilizing these three datasets, a combined OR of 0.85 (95% CI = 0.77–0.93; p = 2.7×10^−4^) was observed for rs2659056.

**Table 3:** Association between *KLK15* shortlisted SNPs and prostate tumour aggressiveness, using a case-case analysis.

SNP	Genotype	Non-aggressive cases (GS<7)	Aggressive cases (GS≥7)	OR (95 CI)^a^	P value
***rs2659058***	TT	118 (46.6)	277 (45.4)		
	CT	108 (42.7)	269 (44.1)	1.01(0.81–1.27)	0.91
	CC	27 (10.7)	64 (10.5)		
***rs3212810***	CC	143 (56.7)	349 (57.2)		
	TC	97 (38.5)	229 (37.5)	1.00(0.78–1.28)	1.00
	TT	12 (4.8)	32 (5.2)		
***rs3745522***	GG	145 (56.2)	353 (57.7)		
	GT	97 (37.6)	218 (35.6)	0.98(0.77–1.24)	0.88
	TT	16 (6.2)	41 (6.7)		
***rs2659056***	TT	111 (44.0)	343 (56.2)		
	TC	118 (46.8)	228 (37.4)	**0.70(0.56–0.89)**	**0.003**
	CC	23 (9.1)	39 (6.4)		
***rs266851***	CC	180 (69.2)	398 (65)		
	CT	74 (28.5)	195 (31.9)	1.22(0.92–1.61)	0.17
	TT	6 (2.3)	19 (3.1)		
***rs2659055***	TT	57 (22.5)	153 (25.7)		
	TC	140 (55.3)	308 (51.7)	0.97(0.78–1.21)	0.80
	CC	56 (22.1)	135 (22.7)		
***rs190552***	TT	151 (59.7)	372 (61.1)		
	CT	89 (35.2)	214 (35.1)	0.88(0.68–1.14)	0.34
	CC	13 (5.1)	23 (3.8)		
***rs266855***	CC	133 (51.6)	297 (48.7)		
	CT	109 (42.2)	253 (41.5)	1.18(0.93–1.48)	0.17
	TT	16 (6.2)	60 (9.8)		
***rs2739442***	GG	98 (38.4)	200 (32.8)		
	GA	114 (44.7)	280 (46)	1.19(0.97–1.46)	0.09
	AA	43 (16.9)	129 (21.2)		
***rs2659053***	GG	83 (32.9)	217 (35.6)		
	GA	117 (46.4)	298 (48.9)	0.86(0.7–1.07)	0.17
	AA	52 (20.6)	94 (15.4)		
***rs2569746***	AA	100 (39.5)	212 (34.8)		
	TA	109 (43.1)	303 (49.8)	1.04(0.84–1.29)	0.71
	TT	44 (17.4)	94 (15.4)		
***rs35711205***	CC	159 (61.2)	381 (62.2)		
	CG	87 (33.5)	211 (34.4)	0.90(0.7–1.16)	0.42
	GG	14 (5.4)	21 (3.4)		

a The result of trend test based on logistic regression adjusted for age as continuous variable.

Bold represents significant p values.

**Table 4:** Association between KLK15 rs2659056 SNP and prostate tumour aggressiveness in five different study groups, using a case-case analysis.

SNP	Controls	Non-aggressive cases (GS<7)	Aggressive cases (GS≥7)	OR (95 CI) Aggressive vs Non-aggressive)	P value
***Australian dataset (n^cases^ = 862, n^control^ = 1375)*** a					
TT	758 (55.1)	111 (44.0)	343 (56.2)		
TC	527 (38.3)	118 (46.8)	228 (37.4)	**0.70 (0.56–0.89)**	**0.003**
CC	90 (6.5)	23 (9.1)	39 (6.4)		
***UK GWAS Stage 1 (n^cases^ = 1232 n^control^ = 1894)*** b					
TT	1166 (61.6)	368 (55.1)	298 (52.8)		
TC	650 (34.3)	250 (37.4)	224 (39.7)	1.06 (0.89–1.26)	0.54
CC	78 (4.1)	50 (7.5)	42 (7.4)		
***UK GWAS Stage 2 (n^cases^ = 2343, n^control^ = 3936)*** b					
TT	2164 (55)	680 (55.3)	606 (54.4)		
TC	1510 (38.4)	478 (38.9)	443 (39.8)	1.03 (0.9–1.17)	0.71
CC	262 (6.7)	71 (5.8)	65 (5.8)		
***UK GWAS Stage 3 (n^cases^ = 3041, n^control^ = 4165)*** b					
TT	2346 (56.3)	771 (52.3)	875 (56.0)		
TC	1536 (36.9)	584 (39.6)	595 (37.9)	**0.87 (0.78–0.98)**	**0.020**
CC	283 (6.8)	119 (8.0)	97 (6.1)		
***CGEMS (n^cases^ = 1148; n^control^ = 1145)*** c					
TT	624 (54.5)	259 (53.0)	359 (54.5)		
TC	443 (38.7)	194 (39.7)	261 (39.6)	0.93 (0.77–1.12)	0.43
CC	78 (6.8)	36 (7.4)	39 (5.9)		
***Combined Results (n^cases^ = 8,626; n^control^ = 12,515)*** c					
TT	7058 (56.4)	2189 (53.2)	2481 (55.0)		
TC	4666 (37.3)	1624 (39.5)	1751 (38.8)	**0.92 (0.86–0.98)**	**0.017**
CC	791 (6.3)	299 (7.3)	282 (6.2)		
***Combined Results with studies selected on heterogeneity test (n^cases^ = 5,051; n^control^ = 6,685)*** c					
TT	3728 (55.8)	1141 (51.5)	1577 (55.6)		
TC	2506 (37.5)	896 (40.5)	1084 (38.2)	**0.85 (0.77–0.93)**	**2.7×10^−4^**
CC	451 (6.7)	178 (8.0)	175 (6.2)		

a The result of trend test based on logistic regression adjusted for age as continuous variable.

b UK dataset adjusted for study group (categorical) and age (continuous.)

c Adjusted for study group and age as categorical variable.

n^cases^ = total no of patients; n^control^ = total no of controls.

## Discussion

In the current study, 12 SNPs were genotyped in 1,011 Australian prostate cancer cases and 1,405 male controls from an initially chosen set of 22 SNPs (7 tag SNPs from the HapMap and 15 SNPs selected on the basis of *in silico* analysis). Two SNPs, rs2659053 and rs35711205, present in the putative promoter region of *KLK15* gene (both upstream of exon “A”) [26], showed evidence of an association with risk of prostate cancer. However, in a recent study, of 1,179 cases and 1,124 control subjects, published by Parikh *et al*, these two SNPs were not found to be significantly associated with risk of prostate cancer from imputed data from the PLCO cohort [27]. Although this might suggest that our findings reflect false-positive associations, to the best of our knowledge these SNPs have not been directly genotyped in previous GWAS [28] [25] or candidate gene association studies focused on the *Kallikrein* locus [19] and hence need replication in a bigger sample set.

The *KLK15* tagSNP rs2659056 was found to be significantly associated with risk of prostate cancer only in the UK GWAS stage 1 dataset, but in no other datasets. This may possibly be due to different patient and control selection criteria. Specifically, the UK GWAS stage 1 controls [24] were selected by design for low PSA (<0.5 ng/ml) and no limitations were placed on case-group PSA values, while stage 2 and stage 3 UK GWAS datasets, demonstrating attenuated risk estimates, had less stringent selection of controls (PSA levels of <10 and requiring a negative prostatic biopsy if the PSA was >4). In addition, QLD and CGEMS samples showing no association with risk had no selection of controls by PSA. In support of this explanation, the control allele frequency in the UK stage 1 dataset differs in comparison to the other datasets (p = 0.0001). Interestingly, we found a significant association of the same SNP with prostate cancer aggressiveness in our QLD study cohort. There was no genotypic association between the rs2659056 SNP and various other clinical markers in healthy men, including serum vasectomy (p = 0.89), and alcohol consumption (p = 0.30), thus these clinical variables are not confounding our results. There was evidence for replication in the UK GWAS stage 3 dataset of more than 3,000 patients from UK and Australia and the CGEMS study of ∼1,000 US patients for the association of the rs2659056 SNP and prostate cancer aggressiveness, but not in the UK GWAS stage 1 and stage 2 datasets, with significant heterogeneity observed across the datasets driven by the UK GWAS stage 1 and stage 2 dataset. This heterogeneity may be explained in part by differences in tumor grading systems by urologists in different countries, as well as by different patient recruitment criteria for the different sample sets - for example, the Australian patient samples were pathology-confirmed patients who presented with symptomatic disease, whilst the UK GWAS stage 1 samples were detected by PSA screening and were also enriched for early onset disease or patients with familial history of prostate cancer. This interesting finding would benefit from further replication in very large consortium sample sets, such as those of PRACTICAL (*Pr*ostate *c*ancer *a*ssociation group *t*o *i*nvestigate *c*ancer *a*ssociated a*l*terations in the genome consortium).

SNP rs2659056 was selected as a HapMap tagSNP, but is located in a gene regulatory region ∼400 bps downstream of a newly identified *KLK15* exon [26]. It was thus assessed for a possible causal effect on transcription factor binding affinities to investigate if it might alter *KLK15* gene expression via this mechanism. The TFSEARCH (http://www.cbrc.jp/research/db/TFSEARCH.html) database indicated that an A to G change in the rs2659056 increases scores for binding of the orphan nuclear receptor RORalpha, which has been shown to be involved in the control of cell growth and differentiation, along with the control of metastatic behavior of androgen-independent prostate cancer cells [29]. Thus, the association of rs2659056 SNP with prostate cancer aggressiveness, if confirmed in larger studies, would prioritize rs2659056 SNP itself as possible causative SNP.

In line with our results, Parikh *et al* recently identified significant associations between *KLK3* SNPs in nonaggressive prostate cancer only [27]. Our results and that of Parikh *et al* suggest that the risk effects observed in the PSA locus may reflect the increased identification of men with clinically insignificant and non-life threatening prostate cancers by the use of PSA for screening of prostate cancer. It is however possible that the *Kallikrein* locus SNPs contribute to PSA levels and prostate cancer independently, and thus further studies are needed to delineate the role of *Kallikrein* locus SNPs in prostate cancer etiology.

In conclusion, this work represents an in-depth study of genetic variation in the *Prostinogen*/*KLK15* gene. Our investigation has made maximum utilization of existing databases and bioinformatic software programs to shortlist SNPs for inclusion in a prostate cancer genetic association study. We identified rs2659056 to be associated with tumor aggressiveness in a QLD sample set and this result was replicated in two large international cohorts. Additional experimental evidence is required to replicate our results and to understand the effects of this variant on the regulation of *KLK15* expression, and its relationship with PSA levels and possible confounders introduced by case and control selection criteria based on PSA levels.

## Materials and Methods

### Ethics Statement

The study protocol was approved by the Human Research Ethics Committees of QUT, QIMR, the Mater Hospital (for Brisbane Private Hospital), the Royal Brisbane Hospital, Princess Alexandra Hospital and the Cancer Council Queensland. All participants gave written informed consent.

### Study Participants

#### Queensland (QLD) prostate cancer cases and controls

QLD prostate cancer cases (N = 1,011) were ascertained from two studies. In the first cross-sectional study, men with prostate cancer were recruited within two years of diagnosis through urologist referrals from three hospitals in Brisbane, Queensland (N = 154, age range 51–87 years) [17]. In the second longitudinal randomized control trial study entitled Prostate Cancer Supportive Care and Patient Outcomes Project (ProsCan): men newly diagnosed with prostate cancer from 26 private practices and 10 public hospitals in Queensland were directly referred to ProsCan at the time of diagnosis by their treating clinician (N = 857, age range 43–88 years) [30]. All cases had histopathologically confirmed prostate cancer, following presentation with an abnormal serum PSA and/or lower urinary tract symptoms. Male controls (N = 1,405) with no personal history of prostate cancer were recruited from two different sources. Male blood donors were recruited through the Australian Red Cross Blood Services in Brisbane (N = 836, age range 18–75 years) [17]. The second control group comprised men randomly selected from the Australian Electoral Roll (voting is compulsory in Australia), age-matched (in 5 year groups; age range 54–90 years) and area-code matched to ProsCan cases (N = 569). Clinical and epidemiologic characteristics of participants are detailed in Table 1.

#### Replication set

Analyses were based on samples genotyped in first and second stages of an UK/Australian GWAS, collected as previously described [24], [28], together with a third stage involving a further 4,574 (3,041 with data on Gleason score) cases and 4,165 controls. Briefly, stage 1 prostate cancer cases (N = 2,017) were from the UK Genetic Prostate Cancer Study (UKGPCS) and were selected on the basis of either a diagnosis at age ≤60 years (N = 1,291) or a first- or second-degree family history of prostate cancer (N = 726). Male controls (N = 2,001) included men aged ≥50 years with a PSA of ≤0.5 ng/ml, geographically matched to the prostate cancer cases selected through the ProtecT study.

Stage 2 comprised prostate cancer cases and controls from the UK and Australia. The former were ascertained through the UKGPCS as above (N = 332) and through a systematically collected series from prostate cancer clinics in the Urology unit at the Royal Marsden NHS Foundation Trust (N = 1,680) over a 14-year period. UK controls were identified through the UKGPCS study (N = 449) and the ProtecT study (limited to those men with a PSA of <10 ng/ml; N = 1,712). Self-reported “non-white” men were excluded. The Australian stage 2 cases were ascertained from three studies: (i) a population based series of prostate cancer cases identified from the Victorian Cancer Registry since 1999, diagnosed at <56 years (Early Onset Prostate Cancer Study (EOPCFS); N = 526); (ii) a population-based case-control study based on cases diagnosed in Melbourne and Perth (Risk Factors for Prostate Cancer Study (RFPCS); N = 594); and (iii) a prospective cohort study of 17,154 men aged 40–69 years at recruitment in 1990–1994 (Melbourne Collaborative Cohort Study (MCCS); N = 190). For RFPCS, cases were identified from the population cancer registries, had histopathologically confirmed prostate cancer (excluding tumors with Gleason scores of less than 5) and were diagnosed at <70 years with sampling stratified by age at diagnosis. Australian stage 2 controls were either recruited as part of the RFPCS study, in which they were identified through the Australian Electoral Roll and frequency matched to the age distribution of the RFPCS cases (N = 509), or were a random sample from the MCCS cohort (N = 760).

Stage 3 samples were selected from UKGPCS as for stage 1 and 2; from Studies of Epidemiology and Risk factors in Cancer Heredity (SEARCH), a case-control study based on region covered by the Eastern UK Cancer Registry and Information Centre (ECRIC); and from the Australian epidemiological studies as in stage 2.

We also included data from the Cancer Genetic Markers of Susceptibility (CGEMS) study, a GWAS of 1,117 prostate cancer cases oversampled for aggressive disease and 1,105 controls, drawn from the European PLCO study (http://cgems.cancer.gov/).

### *KLK15* Sequencing and Genotyping

Methods used for DNA preparation and genotyping have been described previously [18]. Briefly, germline DNA was extracted from peripheral blood using the Qiagen DNA isolation kit for all men recruited in the study. Four primer sets were designed to amplify selected regions chosen from the *in silico* analysis of the putative *KLK15* promoter region. For promoter sequencing, primer sets were designed using NETprimer (http://www.premierbiosoft.com/netprimer/netprlaunch/etprlaunch.html) and purchased from Sigma Proligo (Sigma Proligo, NSW, Australia). Ten ng of germline DNA from 20 aggressive prostate cancer patients was amplified in a 20 uL polymerase chain reaction (PCR) mix optimised for each primer as described previously [26].

SNPs in the Queensland (QLD) dataset was genotyped using iPLEX Gold assays on the Sequenom MassARRAY platform (Sequenom, San Diego, USA), as described previously [18]. Quality control parameters included a combination of cases and controls on each plate, genotype call rates >95%, ≥98% concordance between duplicates (>5% duplicates on each plate), four negative (H_2_O) controls per 384-well plate and Hardy-Weinberg Equilibrium P values>0.05.

Genotyping of the replication sample sets was performed as part of a published genome-wide association study (GWAS) [25], [28]. The Stage 3 genotyping was done using an Illumina Golden Gate Assay (http://www.illumina.com).

### Statistical Analysis

Covariates, including age at diagnosis, screening history and first-degree family history of prostate cancer, were examined to see if such factors changed the risk estimates by ≥10%. After these tests, only age at diagnosis (continuous variable) and study group (as a categorical variable) was included in the final models. Predictive Analytics Software (PASW) Statistics version 17.0.2 (SPSS Inc, Chicago, Illinois) was used for all analyses, unless otherwise specified. Comparisons of genotype distribution and their association with prostate cancer susceptibility and clinical data were performed under co-dominant and linear models, using chi-square and logistic regression analysis, and odds ratios and p were values calculated. Prostate cancer cases with tumor Gleason scores ≥7 were classified as aggressive. For the combined analysis, genotype and phenotype data (disease status, Gleason score, age, family history etc) was obtained for different studies and was analysed as per above after adjusting for study groups and age (as a categorical variable). The extent of heterogeneity across studies was measured by the likelihood ratio test. After applying Bonferroni correction, a p value of <0.004 was considered significant to account for the 12 SNPs studied.

## Supporting Information

Figure S1 Linkage Disequilibrium map generated by Haploview 4.2. Frequency data was generated for the control male individuals and the LD map was plotted. SNPs in bold were found to have frequencies>0.05.
(PDF)

Figure S2 Forest plot showing the association between rs2659056 and prostate tumour aggressiveness in five different study groups, using a case-case analysis.
(PPTX)

Table S1 **SNP selection for the** ***KLK15*** **genetic association analysis with risk of prostate cancer.** SNPs in the *KLK15* gene derived from the HapMap database and those by *in silico* prediction methods were genotyped in male control, and the minor allele frequency (MAF) and HWE were calculated using Haploview 4.2 in healthy males. SNPs in bold were shortlisted for genotyping and association analysis on the basis on LD calculations.
(DOC)

Table S2 **Association between** ***KLK15*** **HapMap Tag and putative functional SNPs and prostate cancer risk in the QLD, UK Stage 1 GWAS and PLCO study groups.**
(DOC)
