# Supplementary material for: Association between Prostinogen (KLK15) Genetic Variants and Prostate Cancer Risk and Aggressiveness in Australia and a Meta-Analysis of GWAS Data
Source: PLoS One. 2011 Nov 23;6(11):e26527. doi: 10.1371/journal.pone.0026527 (PMC3223160; doi:10.1371/journal.pone.0026527)
Supplement: Figure S1 — Linkage Disequilibrium map generated by Haploview 4.2. Frequency data was generated for the control male individuals and the LD map was plotted. SNPs in bold were found to have frequencies>0.05. (PDF) [file pone.0026527.s001.pdf]

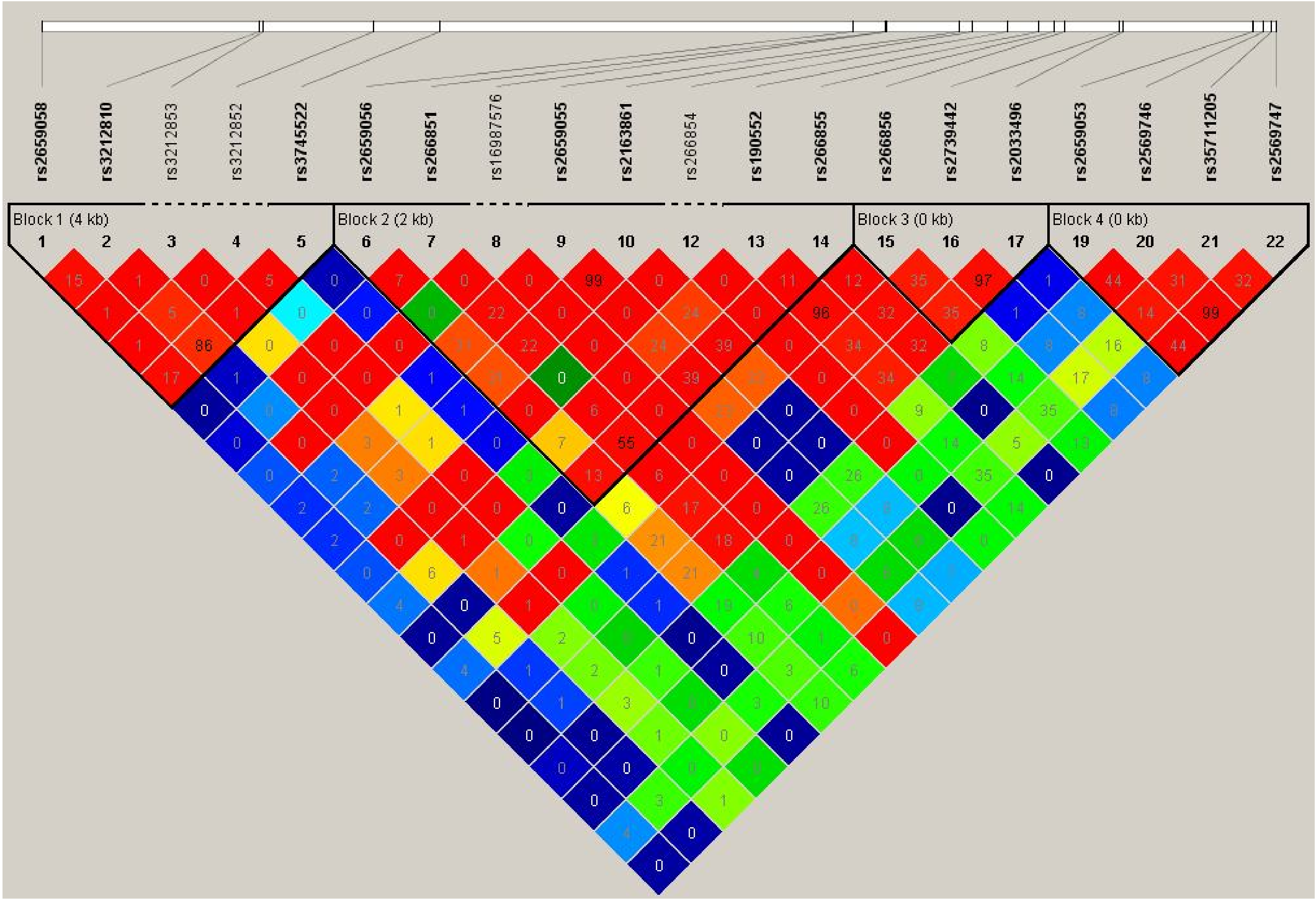

Supplementary Figure 1: Linkage Disequilibrium map generated by Haploview 4.2. Frequency data was generated for the control male individuals and the LD map was plotted. SNPs in **bold** were found to have frequencies>0.05.
