# Supplementary figures and images for: Association between Prostinogen (KLK15) Genetic Variants and Prostate Cancer Risk and Aggressiveness in Australia and a Meta-Analysis of GWAS Data
Source: PLoS One. 2011 Nov 23;6(11):e26527. doi: 10.1371/journal.pone.0026527 (PMC3223160; doi:10.1371/journal.pone.0026527)

## Slide 1
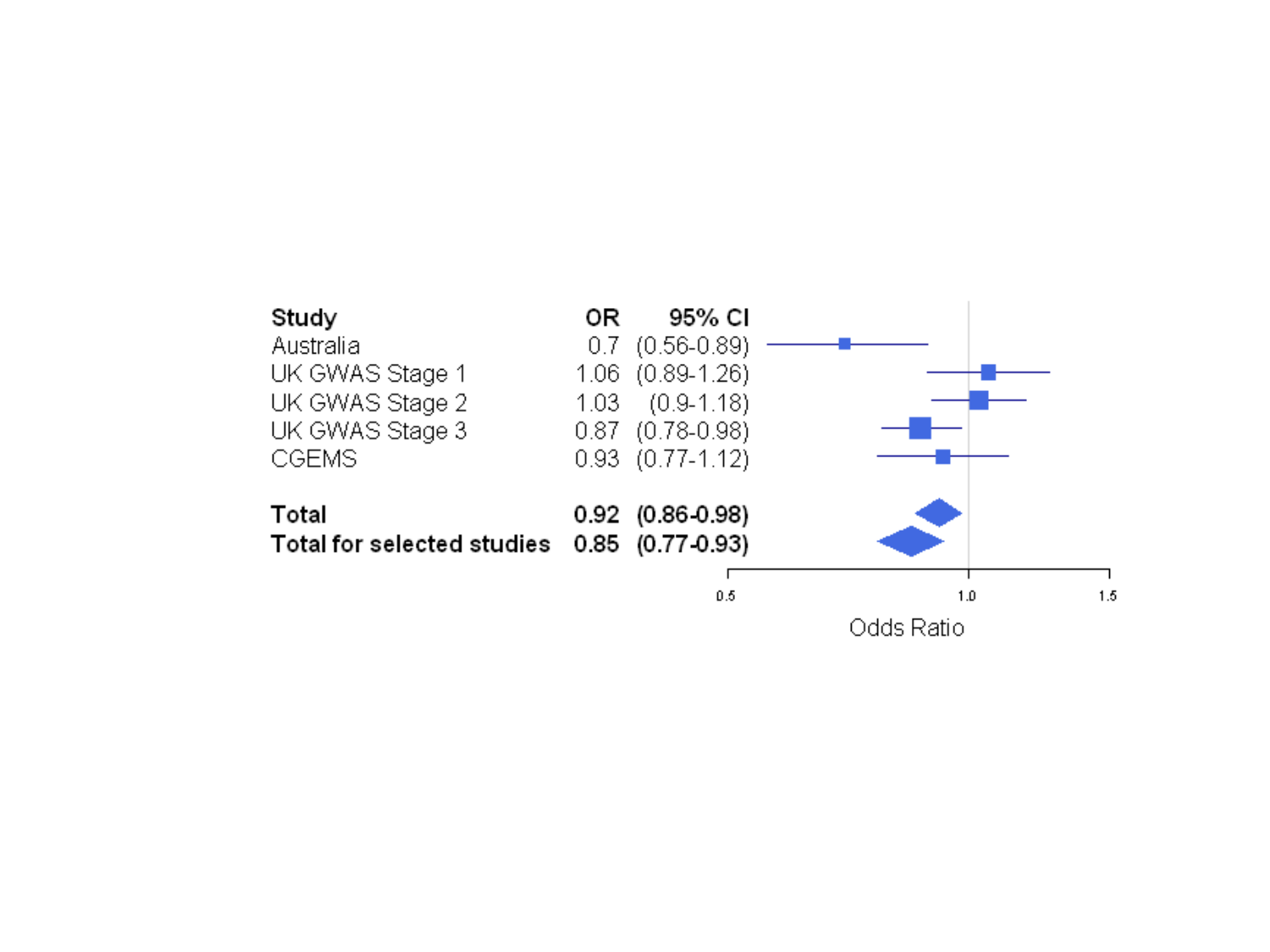

Supplement: Figure S2 — Forest plot showing the association between rs2659056 and prostate tumour aggressiveness in five different study groups, using a case-case analysis. (PPTX) [file pone.0026527.s002.pptx]
