# Supplementary material for: Association between Prostinogen (KLK15) Genetic Variants and Prostate Cancer Risk and Aggressiveness in Australia and a Meta-Analysis of GWAS Data
Source: PLoS One. 2011 Nov 23;6(11):e26527. doi: 10.1371/journal.pone.0026527 (PMC3223160; doi:10.1371/journal.pone.0026527)
Supplement: Table S1 — SNP selection for the KLK15 genetic association analysis with risk of prostate cancer. SNPs in the KLK15 gene derived from the HapMap database and those by in silico prediction methods were genotyped in male control, and the minor allele frequency (MAF) and HWE were calculated using Haploview 4.2 in healthy males. SNPs in bold were shortlisted for genotyping and association analysis on the basis on LD calculations. (DOC) [file pone.0026527.s003.doc]

**Table S1: SNP selection for the *KLK15* genetic association analysis with risk of prostate cancer.** SNPs in the *KLK15* gene derived from the HapMap database and those by *in silico* prediction methods were genotyped in male control, and the minor allele frequency (MAF) and HWE were calculated using Haploview 4.2 in healthy males. SNPs in bold were shortlisted for genotyping and association analysis on the basis on LD calculations.

| **SNP no** | **rsID** | **Position** | **MAF** | **HWpval** | **Rationale** |
| --- | --- | --- | --- | --- | --- |
| 1 | **rs2659058** | 56017918 | 0.331 | 0.09 | **Tagged SNP** from HapMap |
| 2 | **rs3212810** | 56020548 | 0.244 | 0.61 | **Tagged SNP** from HapMap and predicted to fall in miRNA binding site |
| 3 | rs3212853 | 56020606 | 0.037 | 0.29 | Ploymorphic miRNA target site |
| 4 | rs3212852 | 56021941 | 0.019 | 1.00 | Exon-Intron boundary |
| 5 | **rs3745522** | 56022744 | 0.259 | 0.00 | **Tagged SNP** from HapMap |
| 6 | **rs2659056** | 56027755 | 0.257 | 1.00 | **Tagged SNP** from HapMap |
| 7 | **rs266851** | 56028151 | 0.189 | 0.27 | **Tagged SNP** from HapMap |
| 8 | rs16987576 | 56028165 | 0.001 | 1.00 | 2 bp 3' of exon B, Imp for splicing |
| 9 | **rs2659055** | 56029044 | 0.49 | 0.30 | Predicted in silico to affect HRE |
| 10 | rs2163861 | 56029204 | 0.489 | 0.28 | **Tagged SNP** from HapMap |
| 11 | rs266853 | 56029520 | 0.001 | 1.00 | Predicted in silico to affect TFBS and HRE |
| 12 | rs266854 | 56029630 | 0.002 | 1.00 | Predicted in silico to affect HRE |
| 13 | **rs190552** | 56030005 | 0.222 | 0.18 | Predicted in silico to affect TFBS and HRE |
| 14 | **rs266855** | 56030196 | 0.292 | 0.13 | Predicted in silico to affect HRE |
| 15 | rs266856 | 56030318 | 0.225 | 0.11 | **Tagged SNP** from HapMap |
| 16 | **rs2739442** | 56030985 | 0.442 | 0.30 | Predicted in silico to affect TFBS and HRE |
| 17 | rs2033496 | 56031019 | 0.442 | 0.83 | Predicted in silico to affect TFBS and HRE |
| 18 | rs12978902 | 56031056 | 0 | 1.00 | Predicted in silico to affect HRE |
| 19 | **rs2659053** | 56032606 | 0.382 | 0.07 | Predicted in silico to affect TFBS and HRE |
| 20 | **rs2569746** | 56032727 | 0.42 | 0.06 | Predicted in silico to affect TFBS and HRE |
| 21 | **rs35711205** | 56032818 | 0.191 | 0.12 | Novel SNP (reported later in NCBI) |
| 22 | rs2569747 | 56032875 | 0.419 | 0.03 | Predicted in silico to affect TFBS and HRE |

MAF: Minor allele frequency; HWpval: Hardy-Weinberg equilibrium p value; HRE: Hormone Response Element; TFBS: Transcription Factor Binding Site
