# Supplementary material for: Association between Prostinogen (KLK15) Genetic Variants and Prostate Cancer Risk and Aggressiveness in Australia and a Meta-Analysis of GWAS Data
Source: PLoS One. 2011 Nov 23;6(11):e26527. doi: 10.1371/journal.pone.0026527 (PMC3223160; doi:10.1371/journal.pone.0026527)
Supplement: Table S2 — Association between KLK15 HapMap Tag and putative functional SNPs and prostate cancer risk in the QLD, UK Stage 1 GWAS and PLCO study groups. (DOC) [file pone.0026527.s004.doc]

| **Table S2: Association between *KLK15* HapMap Tag and putative functional SNPs and prostate cancer risk in the QLD, UK Stage 1 GWAS and PLCO study groups.** | | | | | | | | | | | |
| --- | --- | --- | --- | --- | --- | --- | --- | --- | --- | --- | --- |
| Qld DATA | | | | | UK Stage 1 GWAS | | | PLCO Cancer Screening Trial (CGEMS) | | | |
| SNP^a^ | Control | Cases | OR (95% CI) | P value^c^ | SNP^b^ | OR (95% CI) | P value^d^ | SNP^b, f^ | OR (95% CI) | p value^e^ | |
| *rs2659058* |  |  |  |  | *rs266114* |  |  | *rs266114* |  |  | |
| TT | 625 (45.7) | 449 (45.1) |  |  | TT |  |  | TT |  |  | |
| CT | 579 (42.3) | 440 (44.2) | 1.06 (0.89-1.27) | 0.59 | CT | 1.18 (1.03-1.36) | 0.009 | CT | 1.18 (0.98–1.41) | 0.09 | |
| CC | 165 (12.1) | 107 (10.7) | 0.93 (0.70-1.22) |  | CC | 1.31 (1.07-1.59) |  | CC | 1.27 (0.97–1.66) |  | |
| *rs3212810* |  |  |  |  | *rs5519* |  |  |  |  |  | |
| CC | 792 (57.6) | 567 (57.1) |  |  | AA |  |  | Imputed | 0.95 (0.83-1.10) | 0.52 | |
| TC | 498 (36.2) | 379 (38.2) | 1.05 (0.88-1.25) | 0.21 | AG | 0.94 (0.82-1.08) | 0.33 |  |  |  | |
| TT | 85 (6.2) | 47 (4.7) | 0.74 (0.51-1.09) |  | GG | 0.85 (0.67-1.07) |  |  |  |  | |
| *rs3745522* |  |  |  |  | *rs3745522* |  |  | *rs3745522* |  |  | |
| GG | 793 (56.8) | 577 (57.4) |  |  | GG |  |  | GG |  |  | |
| GT | 485 (34.8) | 362 (36) | 1.03 (0.87-1.23) | 0.25 | TG | 0.90 (0.79-1.04) | 0.19 | TG | 0.87 (0.73–1.05) | 0.33 | |
| TT | 117 (8.4) | 67 (6.7) | 0.78 (0.56-1.08) |  | TT | 0.85 (0.67-1.07) |  | TT | 0.98 (0.72–1.34) |  | |
| *rs2659056* |  |  |  |  | *rs2659056* |  |  | *rs2659056* |  |  | |
| TT | 758 (55.1) | 532 (53.5) |  |  | TT |  |  | TT |  |  | |
| TC | 527 (38.3) | 393 (39.5) | 1.11 (0.93-1.32) | 0.41 | TC | 1.27 (1.11-1.45) | 5.45x10^-7^ | TC | 1.01 (0.85–1.20) | 0.94 | |
| CC | 90 (6.5) | 70 (7.0) | 1.16 (0.83-1.62) |  | CC | 2.01 (1.50-2.68) |  | CC | 0.95 (0.68–1.33) |  | |
| *rs266851* |  |  |  |  | *rs266851* |  |  | *rs266851* |  |  | |
| CC | 917 (66.1) | 673 (66.8) |  |  | CC |  |  | CC |  |  | |
| CT | 414 (29.8) | 307 (30.5) | 1.03 (0.86-1.23) | 0.15 | TC | 1.05 (0.92-1.21) | 0.48 | TC | 0.92 (0.76–1.10) | 0.37 | |
| TT | 56 (4.0) | 27 (2.7) | 0.64 (0.40-1.02) |  | TT | 1.20 (0.86-1.67) |  | TT | 1.24 (0.79–1.96) |  | |
| *rs2659055* |  |  |  |  | *rs2163861* |  |  | *rs2163861* |  |  | |
| TT | 364 (26.7) | 252 (25.6) |  |  | CC |  |  | CC |  |  | |
| TC | 663 (48.7) | 515 (52.3) | 1.15 (0.94-1.40) | 0.17 | TC | 0.86 (0.74-1.01) | 0.16 | TC | 0.95 (0.78–1.17) |  | |
| CC | 335 (24.6) | 217 (22.1) | 0.96 (0.76-1.22) |  | TT | 0.88 (0.73-1.06) |  | TT | 0.90 (0.71–1.13) | 0.66 | |
| *rs190552* |  |  |  |  | *rs266850* |  |  | *rs266850* |  |  | |
| TT | 842 (61.1) | 584 (58.6) |  |  | AA |  |  | AA |  |  | |
| CT | 458 (33.3) | 366 (36.7) | 1.16 (0.97-1.38) | 0.13 | AG | 1.05 (0.90-1.21) | 0.75 | AG | 1.00 (0.83–1.21) | 0.68 | |
| CC | 77 (5.6) | 46 (4.6) | 0.83 (0.57-1.22) |  | GG | 0.92 (0.60-1.41) |  | GG | 1.26 (0.75–2.11) |  | |
| *rs266855* |  |  |  |  |  |  |  |  |  |  | |
| CC | 703 (50.8) | 499 (49.8) |  |  | ND |  |  | Imputed | 1.09 (0.88-1.34) | 0.425 | |
| CT | 549 (39.7) | 419 (41.8) | 1.07 (0.90-1.28) | 0.38 |  |  |  |  |  |  | |
| TT | 131 (9.5) | 84 (8.4) | 0.87 (0.65-1.18) |  |  |  |  |  |  |  | |
| *rs2739442* |  |  |  |  |  |  |  |  |  |  | |
| GG | 439 (32.0) | 333 (33.4) |  |  | ND |  |  | Imputed | 1.02 (0.86-1.20) | 0.825 | |
| GA | 656 (47.8) | 462 (46.3) | 0.95 (0.79-1.15) | 0.81 |  |  |  |  |  |  | |
| AA | 278 (20.2) | 202 (20.3) | 0.93 (0.74-1.18) |  |  |  |  |  |  |  | |
| *rs2659053* |  |  |  |  |  |  |  |  |  |  | |
| GG | 542 (39.5) | 348 (35.0) |  |  | ND |  |  | Imputed | 1.02 (0.83-1.25) | 0.839 | |
| GA | 615 (44.9) | 486 (48.8) | 1.25 (1.04-1.50) | 0.050 |  |  |  |  |  |  | |
| AA | 214 (15.6) | 161 (16.2) | 1.19 (0.92-1.52) |  |  |  |  |  |  |  | |
| *rs2569746* |  |  |  |  |  |  |  |  |  |  | |
| AA | 480 (34.9) | 346 (34.7) |  |  | ND |  |  | Imputed | 0.92 (0.75-1.13) | 0.418 | |
| TA | 635 (46.2) | 492 (49.4) | 1.05 (0.87-1.26) | 0.13 |  |  |  |  |  |  | |
| TT | 259 (18.9) | 158 (15.9) | 0.83 (0.65-1.06) |  |  |  |  |  |  |  | |
| *rs35711205* |  |  |  |  |  |  |  |  |  |  | |
| CC | 914 (66.0) | 618 (61.3) |  |  | ND |  |  | Imputed | 1.16 (0.90-1.49) | 0.253 | |
| CG | 410 (29.6) | 350 (34.7) | 1.27 (1.06-1.52) | 0.027 |  |  |  |  |  |  | |
| GG | 60 (4.3) | 40 (4.0) | 0.96 (0.63-1.46) |  |  |  |  |  |  |  | |
| ^a^SNP identifier based on NCBI dbSNP; SNPs are included in the region of the *KLK15* gene including 2 kb of transcription start sites | | | | | | | | | | |  |
| ^b^Data on surrogate SNP (r^2^>0.8) is shown if not available on the original SNP. | | | | | | | | | | |  |
| ^c^The result of 2-d.f. test based on logistic regression in the Queensland study adjusted for age as continuous variable.  ^d^The result of 2-d.f. test based on logistic regression in the United Kingdom study. | | | | | | | | | | |  |
| ^e^The result of 2-d.f. test based on logistic regression in the PLCO study adjusted for age in five-year intervals, study center, and three eigenvectors to control population stratification in an incident density sampling strategy. | | | | | | | | | | |  |
| ^f^Imputed from 1000 Genomes project data and PLCO genotyped data, where actual genotype data not available, [27]; allelic OR and p values are presented. | | | | | | | | | | |  |
| ND: Data not available | | | | | | | | | | |  |
